# Attenuated Duck Hepatitis A Virus Infection Is Associated With High mRNA Maintenance in Duckling Liver *via* m6A Modification

**DOI:** 10.3389/fimmu.2022.839677

**Published:** 2022-06-09

**Authors:** Liping Wu, Weili Quan, Yi Zhang, Mingshu Wang, Xumin Ou, Sai Mao, Di Sun, Qiao Yang, Ying Wu, Yaxun Wei, Renyong Jia, Shun Chen, Dekang Zhu, Mafeng Liu, Xinxin Zhao, Shaqiu Zhang, Juan Huang, Qun Gao, Bin Tian, Anchun Cheng

**Affiliations:** ^1^ Institute of Preventive Veterinary Medicine, Sichuan Agricultural University, Chengdu, China; ^2^ ABLife BioBigData Institute, Wuhan, China; ^3^ Center for Genome Analysis, ABLife, Inc., Wuhan, China

**Keywords:** duck hepatitis A virus, m6A modification, liver, duck viral hepatitis, virulence

## Abstract

Host translation is generally modulated by viral infection, including *duck hepatitis A virus* (DHAV) infection. Previously, we reported that cellular protein synthesis in a cell model of duck embryo fibroblasts is significantly inhibited by DHAV infection but not viral proteins, suggesting that an important viral-host interaction occurs at the translational level. In this study, we aim to further understand the impact of DHAV virulence on cellular N6-methyladenosine (m6A) modification, which is essential to a wide variety of RNA biological processes, such as mRNA stabilization and translation. Using m6A antibody-based immunoprecipitation, m6A-seq, and LC–MS/MS, we observed that m6A-modified mRNA exists in both virulent and attenuated DHAV-infected duckling livers. Importantly, m6A levels in mRNA were much higher in attenuated DHAV-infected livers compared with virulent DHAV-infected livers, suggesting virulence-dependent regulation of m6A modification. Analysis of modification motifs indicated that GAAGAAG is the most enriched motif. Combined m6A-seq and RNA-seq data analysis indicated a generally positive correlation between m6A and mRNA expression levels in DHAV-infected duckling livers. GO analysis of genes with decreased or increased m6A levels showed that these genes were enriched in various terms, including oxidation–reduction processes and antiviral immune responses. Collectively, our work reveals DHAV virulence-dependent coordination between m6A modification and mRNA expression in duckling livers.

## Introduction

Duck virus hepatitis, a typical disease caused by duck hepatitis A virus (DHAV), is highly fatal to ducklings (1). Liver damage is the main consequence. To prevent such a disease, vaccination of ducklings with a live attenuated vaccine against DHAV can provide effective immune protection. The vaccine is generated by a series of passages of isolated duck virulent strains in a chicken host. Our previous study reported that the host translation system may force the virulent duck strain to adapt to the chicken host alongside changes in viral codon usage, consequently yielding the successful development of the DHAV attenuated strain (Ou et al., 2018). The virulent DHAV strain can affect overall mRNA translation in infected duckling cells (2, 3). However, the differential translational regulation in the duckling host caused by virulent and attenuated DHAV strains is largely unknown.

During infection, many viruses can inhibit host mRNA translation but not viral RNA translation (4). This mechanism is mechanically related to host mRNA stabilization, cleavage of poly-A binding protein (5), phosphorylation of eIF2alpha (4), induction of stress granules, and tRNA remodelling (4–6). The stabilization of cellular mRNA is essentially regulated by RNA modification, especially m6A modification (7; 8). RNA N6-methyladenosine (m6A) modification is a conserved posttranscriptional modification in mammals that regulates many fundamental aspects of RNA biology, including transcription, splicing, and decay (8). However, this modification has yet to be reported in duck hosts, particularly virulence-dependent regulation of m6A modification. In mammals, this modification is added by the m6A methyltransferase METTL3 (9). Intriguingly, ducks lack this gene, suggesting the existence of an unknown m6A “writer” in ducks.

Recently, it has been reported that m6A plays an important role in viral replication (Hao et al., 2018; 10, 11) and affects cellular RNA metabolism and viral infection (12; 13; 11, 14–19). Transcripts from both DNA and RNA viruses can be methylated and m6A in viral RNA has either proviral or antiviral functions that may be dependent on the viral virulence and host immune situation (7, 14, 19). In contrast, several viruses can alter m6A modifications in cellular mRNAs (13, 20). The role of m6A in cellular mRNA during viral infection is still not well understood due to the difficulties in accurately and quantitatively mapping modification. However, no data on transcriptomic m6A changes in host mRNA after DHAV infection with different virulence strains or the functional consequences of m6A modification have been reported.

To address the above questions, m6A antibody-based immunoprecipitation, m6A-seq, and LC–MS/MS were used to enrich, profile, and quantify the transcriptome-wide m6A modifications in duckling livers and investigate the effect of DHAV virulence on m6A modification. We provide evidence that m6A methylation occurs in duckling liver and the most enriched methylation site is GAAGAAG, which is distinct from the mammalian m6A motif. Importantly, high m6A modification of mRNAs was maintained in DHAV-attenuated strain-infected livers, suggesting that an mRNA stabilization effect was induced by the attenuated strain. Specifically, comparative analysis of attenuated and virulent DHAV-infected duckling livers showed that the m6A level in mRNAs was significantly reduced in livers that were infected by the virulent strain. m6A methylation is strongly coupled with the virulence-regulated mRNA level and the genes under this coupled regulation were distinctly associated with antiviral immune response and stress response. Our results indicate that m6A mRNA methylation is a crucial posttranscriptional modification in duckling liver responding to the virulence of different DHAV infections.

## Results

### DHAV Virulence Regulates Liver Damage in Ducklings

**Figure 1:**
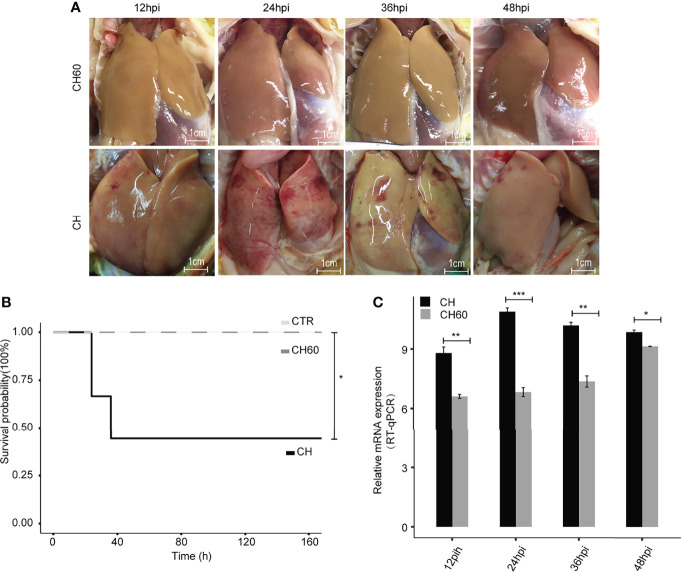
DHAV virulence contributes to the clinical manifestations of infected ducklings. **(A)** Representative images of liver lesions in ducklings. Significant liver haemorrhage is observed after infection with virulent DHAV (CH strain) and corresponding attenuated DHAV (CH60 strain). Scale bar is equal to 1 cm. **(B)** The survival probability of virulent DHAV-attenuated DHAV-infected ducklings (n = 10). Ducklings injected with physiological normal saline served as a control group. Virulent DHAV significantly reduces the survival probability. **(C)** The relative virus titer, CH strain, and CH60 strain in the liver are quantified by RT–qPCR. The liver viral load in ducklings infected with virulent DHAV is significantly increased compared with ducklings infected with attenuated DHAV. Student’s unpaired t test was used for statistical analysis. *P < 0.05, **P < 0.01, ***P < 0.001. Data are displayed as the mean ± SD with 3 biological replicates and 3 technical replicates.

Artificial adaptation of a virulent virus from a native host to a nonnative host has been widely used for vaccine development, such as the duck-to-chicken-embryo DHAV vaccine (defined as attenuated DHAV in context). Our previous study partially revealed that nonnative hosts (i.e., chicken embryos) may force infected duck-isolated DHAV (defined as virulent DHAV in context) to adapt to the chicken host after 60 generations of passaging, resulting in large numbers of synonymous mutations in the DHAV genome (21). In clinical practice, ducklings vaccinated with this type of vaccine exhibit potent immune protection without significant liver pathology compared to the duck virulent DHAV strain (**Figure 1A**). Supportively, ducklings challenged by attenuated DHAV showed 100% survival; however, virulent DHAV was highly lethal and caused 60% death in ducklings (**Figure 1B**). Consistent with our previous study, liver biopsy indicated that liver damage of ducklings infected by attenuated DHAV is minimal (22). Conversely, ducklings challenged by virulent DHAV show evidence of severe liver failure syndrome, such as haemorrhage, as well as other cytopathologies, such as hepatocyte apoptosis and vacuolization degeneration in our previous study and in this study (21, 23) (**Figure 1A**). One possible explanation is that virulent DHAV can robustly replicate in its native host, whereas attenuated DHAV cannot. Supportively, viral RNA qRT–PCR quantification indicated that virulent DHAV replicates much more efficiently in liver tissues than attenuated DHAV (**Figure 1C**). Collectively, this evidence indicates that the virulence of DHAV essentially regulates liver damage.

### Attenuated DHAV Maintains High m6A Levels of mRNAs in Infected Duckling Liver

**Figure 2:**
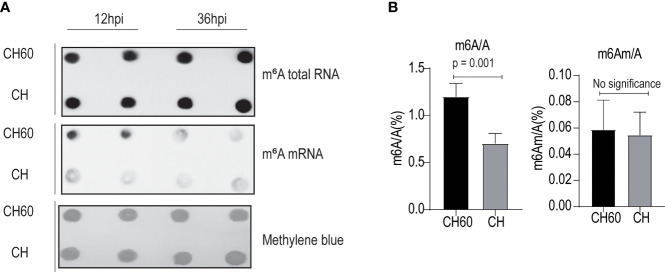
Attenuated DHAV maintains high m6A levels in duckling liver. **(A)** Quantification of m6A modification in total RNAs and mRNAs from duckling livers using m6A antibody-based immunoblotting. Methylene blue staining served as the loading control. Representative dot blot images at 12 hpi and 36 hpi are displayed. m6A-modified mRNA is present at greater levels in the CH60-infected group compared with the CH-infected group (middle panel). **(B)** A more sensitive LC–MS/MS approach was used to further quantify m6A RNA (m6A/A) and m6A mRNA (m6Am/A) in duckling livers infected by a virulent CH strain and attenuated CH60 strain. The virulent CH strain significantly reduces m6A modification levels in the poly-A tailed mRNA pool. However, m6Am (an m6A-resembling modification) modification levels remain similarly low in both groups. Data are displayed as the mean ± SD with 4 replicates.

Virulence or viral infectivity is an indicator of a viral phenotype and seems to regulate many biological processes that contribute to viral pathogenesis. Viral gene expression is largely dependent on the cellular translation machinery. By favouring mRNA translation from the host to the virus, this process mechanically contributes to viral virulence (24). To gain an in-depth understanding of how cellular mRNA dynamically responds to DHAV with different levels of virulence (CH60 versus CH strain), we performed a transcriptome study that focused on m6A-modified mRNAs and features of m6A distribution due to their strong nexus to mRNA translation and stabilization (**Supplementary Table 1**). To specifically sequence m6A-modified mRNAs, total RNA and poly(A)+RNAs that contain m6A modifications were separately enriched using a m6A antibody and poly(A) enrichment kit and subsequently quantified using an immune dot blot assay (**Figure 2A**). We found that m6A levels in total RNA from virulent and attenuated DHAV-infected livers are similar but the m6A levels in poly(A)+ RNA differ (**Figure 2A**). The discrepancy may be caused by a low level (1~5%) of poly(A)+RNA among total RNA that decreases the sensitivity of this approach. Importantly, the m6A level in poly(A)+ RNA (equivalent to mRNAs) of the attenuated DHAV-infected liver seems much higher than that of the virulent DHAV (**Figure 2A**, middle panel). Total m6A levels in mRNAs were further quantified using a more sensitive approach, namely, liquid chromatography/mass spectrometry (LC/MS). The results confirmed high m6A levels in poly(A)+RNAs from attenuated DHAV-infected livers (**Figure 2B**). However, the levels of a similar m6A modification (N6,2′-O-dimethyladenosine, m6Am) in the poly(A)+RNAs remained similar in these two groups at less than 0.06% (**Figure 2B**).

### Features of m6A Distribution and Density Regulated by Virulent and Attenuated DHAV

**Figure 3:**
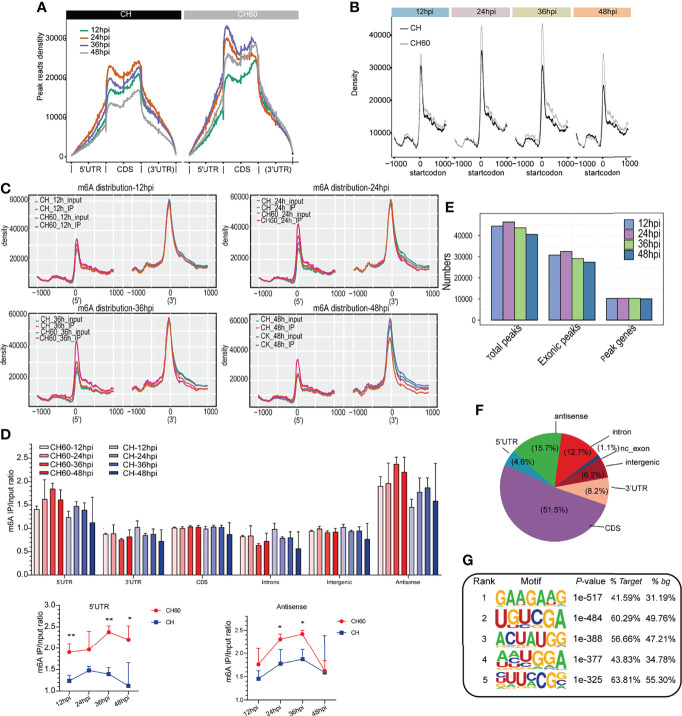
Dynamic density change, distribution, and modification motif of m6A mRNA during infection. **(A)** Enrichment analysis of IP reads around start and stop codons in duckling liver infected with virulent CH and attenuated CH60 strains. During infection, the m6A peak read density in livers infected with the attenuated CH60 strain is much higher than that in livers infected with the virulent CH strain. Each time point after infection is indicated by a different colour. **(B–C)** This m6A modification in attenuated CH60-infected livers is dominantly distributed near the start codon position and near the 5’UTR. **(C)** The m6A distribution of input and IP samples among all samples is analyzed. Inputs and IPs of CH60- or CH-infected livers are displayed in different colours. The m6A density distribution at different infection times is displayed. **(D)** Gene region analysis indicated that the 5’UTR and antisense region are highly modified by m6A (lower panel). Three biological replicates of both m6A IP samples and Input samples were used to calculate m6A IP/Input ratio. The P values were determined using Student’s unpaired t test. *P < 0.05, **P < 0.01. **(E)** The number of peaks and genes with m6A modification detected in both DHAV CH60- and CH strain-infected livers. The m6A modification is highly enriched in the exonic region. **(F)** Distribution of m6A modification in different gene regions. The entire span of a gene, including the 5’UTR, coding sequence (CDS), 3’UTR, and certain noncoding exons, can be modified by m6A. **(G)** The top five enriched m6A motifs are listed and detected by HOMER. As shown, GAAGAAG was the top m6A-modified motif in both the CH60- and CH strain-infected groups.

To further explore the common features of the transcriptomic distribution of m6A, we investigated changes in the distribution of m6A reads and peaks in the gene functional elements of the entire transcriptome. This analysis also integrated all m6A-seq data derived from both virulent and attenuated DHAV-infected duckling livers during the whole course of infection (12 hpi to 48 hpi). Metagene analysis shows that most m6A peak reads were distributed in the CDS region, 5’UTR, and 3’UTR of mRNAs (**Figure 3A** and **Supplementary Figure 1)**, and a high read density is noted in the attenuated DHAV-infected samples compared with virulent DHAV-infected samples (**Figure 3A**), particularly near the start codon from all time points after infection (**Figures 3B–C**). Comparative analysis indicates that the 5’UTR of attenuated CH60-infected samples was significantly modified by m6A (**Figure 3D**). The existence of m6A modifications in the antisense region is interesting but remains biologically unclear (**Figure 3D** and **Supplementary Figure 2**). For example, the tRNA-Ala-UGC-4 antisense fragment is highly modified by m6A and other tRNA antisense fragments are also highly modified (**Supplementary Figure 3**). The calculation of m6A peaks indicated that the m6A modification is highly enriched in the exonic region (ratio= 44487/55519 = 80.12%), a constitutive region of mRNA (**Figure 3E**). Further analysis indicates that entire regions of an mRNA or premRNA, such as the 5’UTR, CDS, and 3’UTR, as well as minimal noncoding exons and introns can be modified by m6A (**Figure 3F** and **Supplementary Figure 2**). HOMER programming was used to understand the common m6A modification motif. We found that GAAGAAG was the top m6A-modified motif in both infected groups (**Figure 3G**). Collectively, these results support that DHAV virulence regulates the mRNA m6A modification level in infected duckling livers.

### DHAV Virulence Coordinates m6A and Expression Levels of Genes Associated With the Oxidation–Reduction Process, Viral Defence Response, and Immune Response

**Figure 4:**
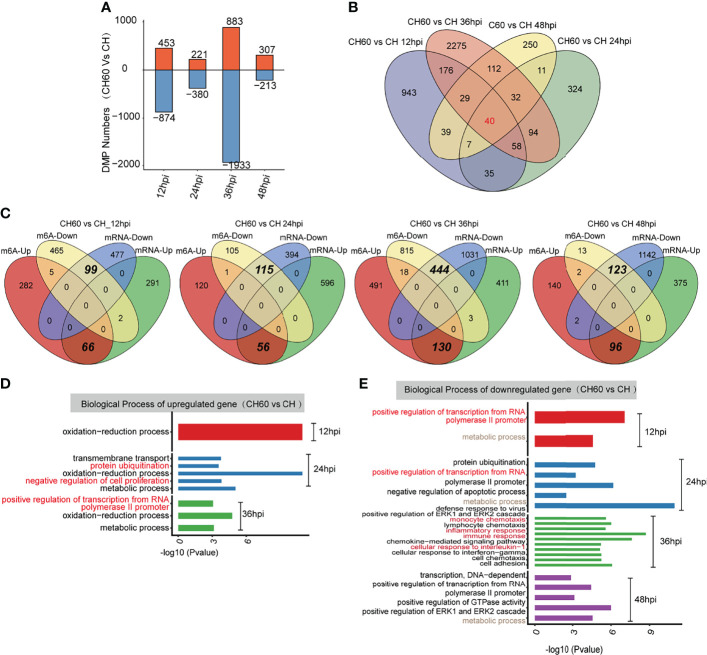
Functional analysis of coding genes with altered m6A peaks between virulent (CH) and attenuated (CH60) DHAV-1 duckling livers. **(A)** The number of hypermethylated (up, red colour) and hypomethylated (down, blue colour) m6A peaks between the DHAV CH60 and CH strains at 12 h, 24 h, 36 h, and 48 h postinfection with DHAV-1. **(B)** Venn diagrams showing the number of altered m6A peaks between DHAV CH60- and CH-stained livers at each time point. Some m6A-modified genes are further validated by qRT–PCR (**Supplementary Figures 5–7)**. **(C)** Venn diagrams showing the number and relationship of m6A peaks and mRNA expression between CH60 and CH at each time point. (**D**, upregulated genes and **E**, downregulated genes) GO biological terms of genes with altered m6A peaks and mRNA expression levels between the DHAV CH60 and CH strains at each time point.

To further explore the potential biological consequence of m6A alteration, we focused on transcripts containing at least one m6A peak showing alteration between CH60 and CH samples at each postinfection time point. This comparison allowed us to study the genes with mRNA modifications that could be related to DHAV virulence. Transcripts with m6A peaks at the same genome position identified in all replicates of DHAV CH- or CH60-stained livers at each time point were used for further analysis. The greatest differences are observed in the CH60 vs. CH comparison (36 hpi) (**Figure 4A**). In particular, for the CH60 vs. CH (12 hpi) and CH60 vs. CH (36 hpi) comparisons, the number of hypomethylated m6A peaks (874 and 1,933 m6A peaks for 12 hpi and 36 hpi, respectively) is approximately one-fold greater than that of hypermethylated m6A peaks (453 and 883 m6A peaks for 12 hpi and 36 hpi, respectively) in CH60 samples (**Figure 4A**). Among these virulence-related genes, a total of 40 common peaks are shared by all four infection time points, whereas 943, 324, 2,275 and 250 specific peaks exclusively appear in CH60 and CH at 12 hpi, 24 hpi, 36 hpi and 48 hpi, respectively (**Figure 4B**). These results suggest that the mRNA methylation of genes may be involved in the response to DHAV virulence in duckling liver.

We next integrated m6A-Seq and RNA-Seq (input) data to explore whether the extent of m6A methylation was associated with mRNA levels of differentially expressed genes (DEGs). Based on RNA-seq, no significant difference in the expression of potential m6A writers and erasers is noted (**Supplementary Figure 4**). In the comparisons of CH60 vs. CH, 359, 652, 544, and 471 upregulated DEGs were identified for 12 hpi, 24 hpi, 36 hpi and 48 hpi, respectively, of which 66, 56,130, and 96 genes were hypermethylated (**Figure 4C**). The hypermethylation of certain biologically important genes, such as suppressor of cytokine signalling 3 (SOCS3), pyruvate dehydrogenase E1 subunit alpha 1 (PDHA1) and cysteine dioxygenase type 1 (CDO1), was validated by qRT–PCR (**Supplementary Figures 5–7** and **Supplementary Table 2**). This finding is consistent with our previous study showing that SOCS3 mRNA is hypermethylated, but the methylated nucleotides remain unclear (22). We now understand that m6A represent such a modification. For comparisons of CH60 vs. CH at each time point, 99, 115, 444 and 123 downregulated genes were hypomethylated at 12 hpi, 24 hpi, 36 hpi and 48 hpi, respectively (**Figure 4C**). These results demonstrate that a number of the DEGs showed m6A alterations in their mRNAs. Notably, in all comparisons, the downregulated DEGs are almost exclusively coincidental with hypomethylated m6A and the upregulated DEGs are coincidental with hypermethylated m6A (**Figure 4C**). These results strongly suggest that m6A may be an important modification regulating gene expression in the duckling liver.

GO analysis of upregulated DEGs with hypermethylated m6A for each comparison show that these genes are commonly enriched in the oxidation–reduction biological process **(** **Figure 4D** **)**. The downregulated DEGs with hypomethylated m6A for each group are enriched in terms including defence response to the virus (CH60 vs. CH at 24 hpi), immune response (CH60 vs. CH at 36 hpi), and positive regulation of GTPase activity (CH60 vs. CH at 48 hpi) (**Figure 4E**). These results further show that m6A methylation is actively involved in regulating the duckling liver response to DHAV virulence.

### mRNA Translation, Protein Metabolism, and tRNA Biology May be Collaboratively Regulated by Attenuated DHAV-Mediated m6A Modification

**Figure 5:**
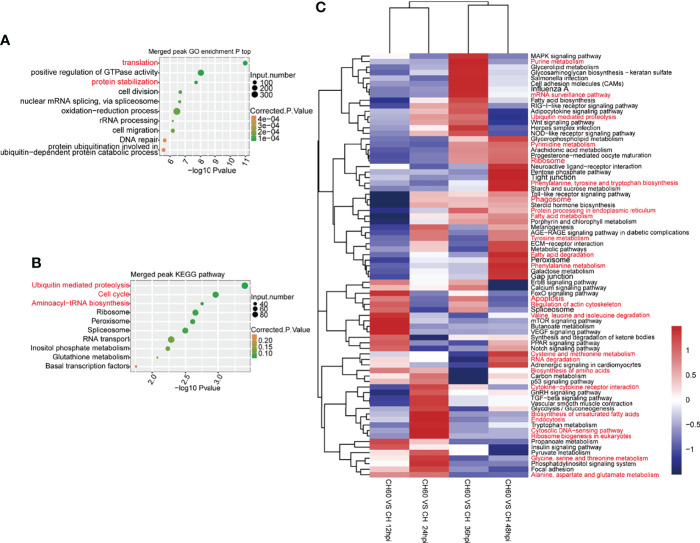
m6A-related RNA–protein biology was coordinately and dynamically regulated by DHAV virulence. **(A)** Commonly regulated GO biological processes in both CH60- and CH strain-infected livers. Basic biological processes, such as mRNA translation, positive regulation of GTP activity, and protein stabilization, are concurrently regulated by both groups. **(B)** A similar analysis was used to identify commonly regulated KEGG pathways in which proteolysis, ribosome, and aminoacyl-tRNA biosynthesis were enriched. This is partially in line with certain m6A modification on tRNA related fragments in sense and antisense, despite lacking statistical significance between both two groups (**Supplementary Figure 3**). **(C)** The dynamic regulation of biological processes of m6A-modified genes was analyzed. During infection, virulence-related mRNA translation and protein metabolism-related pathways were differentially regulated at different infection stages, such as nucleotide metabolism, ribosome, and RNA degradation for mRNA translation, amino acid metabolism, ribosome biosynthesis, and protein processing for protein metabolism.

As confirmed in our previous study, DHAV virulence significantly regulates host protein expression (Liu et al., 2021). This phenomenon is mechanically related to RNA biology, particularly mRNA-protein biology. Cellular functions, including the above-mentioned oxidation–reduction process and immune responses, are regulated by mRNA-to-protein biology as well as tRNA biology, which is involved in mRNA-directed protein synthesis (25). We integrated GO analysis and KEGG pathway analysis to understand the regulatory network that was coordinately regulated by m6A. In both DHAV CH60- and CH strain-infected livers, GO analysis shows that m6A peak-associated genes were commonly enriched in various terms, including translation, positive regulation of GTP activity, and protein stabilization (**Figure 5A**). This analysis was consistent with the results of the KEGG pathway analysis, in which proteolysis, ribosome, and aminoacyl-tRNA biosynthesis are coordinately regulated with the GO analysis (**Figure 5B**). These essentially regulated RNA–protein biology mechanisms and pathways are co-ordinately enriched in both CH60 and CH strain-infected livers.

However, virulence-related regulation of KEGG pathways may change in a time-dependent manner. During infection from 12 hpi to 48 hpi, key pathways that control RNA–protein biology were coordinately and dynamically regulated, such as nucleotide metabolism, ribosome and RNA degradation for RNA biology, amino acid metabolism, ribosome biosynthesis, and protein processing for protein biology (**Figure 5C**). This result suggests that during infection, virulence-related mRNA translation and amino acid metabolism are dynamically regulated at different infection stages, suggesting a possible time-dependent supply of amino acids. Collectively, this evidence suggests that mRNA translation, protein metabolism, and tRNA biology are collaboratively regulated by DHAV virulence through the regulation of m6A mRNA modification.

## Discussion

Human and duck hepatitis A viruses have profound effects on pathophysiology and regulate cellular gene expression at the transcriptional level (22, 26). m6A is the conserved posttranscriptional modification of cellular mRNA in mammalian cells (Lichinchi et al., 2016; 8). Herein, we have identified a m6A modification profile, specific m6A modification sites, and the associated biological consequences in cellular mRNAs in duck livers after challenge with avian hepatotropic DHAV. In our study, m6A modification was mainly located at the start codon near the 5’UTR but was also found in other gene regions, such as the 3’UTR, noncoding exons, and introns (**Figure 3F**). The density of m6A modification around the start codon was significantly related to DHAV virulence (**Figure 3B**). This profile was different from that in humans, pigs, mice, and plants, in which m6A was significantly enriched around the stop codon (27; 28; 29, 30). The distribution of the m6A peak in the CDS is also possible, as observed in *Trypanosoma brucei* (31) and *Bombyx mori* (32) for example. These findings represent the first report of duck m6A modification of mRNA molecules.

The duck genome does not encode the METTL3 gene, which is homologous to the m6A methyltransferase in mammals (9). Despite the absence of this gene, we noticed that other less studied methyltransferases, such as METTL6, METTL9 and METTL21A, were relatively abundant at the mRNA level, suggesting an alternative role for these genes responsible for m6A modification, but more data are needed (**Supplementary Figure 4**). In mammalian cells, the most enriched motif is GGACU (27, 33; Meyer et al., 2012; 34). However, the most enriched m6A motif in duckling liver mRNA was GAAGAAG **(** **Figure 3G**). Notably, *Escherichia coli* (35), *T. brucei* (31)*, and Arabidopsis thaliana* (36) also showed specific patterns in the m6A consensus motif. This evidence indicated that the m6A motif varied among different host species and the m6A modification occurred at the GAAGAAG motif in duck. However, the methyltransferase that catalyses m6A modification in duck is currently unknown. The high mRNA abundance of methyltransferases METTL6, METTL9, and METTL21A suggests that these methyltransferases may be alternatively used for m6A modification (**Supplementary Figure 4**). Further assessment of duck METTL activity at the unique RNA motifs identified in this study would be interesting.

We further explored whether m6A modification affects mRNA expression levels, leading to the finding that virulence-regulated changes in m6A modification and mRNA expression are strongly coupled. The direction of the methylation change is coincidental with that of the mRNA level change. This finding is consistent with the finding that m6A modification occurs cotranscriptionally (Knuckles et al., 2017; 37), and cotranscriptional loading of Dgcr8 and Mettl3 to heat-shock genes in murine embryonic stem cells marks these mRNAs for subsequent RNA degradation (Knuckles et al., 2017). m6A-mediated mRNA decay may occur *via* bystander mechanisms, for example, by recruitment of the reader proteins YTHDF2 (38) and YTHDC1 (39). It is interesting to investigate the impact of m6A modification on mRNA expression *in vitro*; however, we have demonstrated that m6A modification of mRNA is significantly regulated by DHAV virulence.

Cellular biology is largely regulated by RNA biology, especially mRNA translation and protein metabolism. These essential biological processes are believed to be collaboratively regulated by different stimuli, including viral infection. In this study, following the finding of DHAV virulence-dependent m6A modification, we used our high confidence m6A-sequencing data to perform a comprehensive association study (40). Genes with m6A modifications are highly relevant to mRNA translation and protein metabolism. m6A itself may not represent a simple proviral or antiviral mechanism during infection. Rather, this modification distinctly modulates specific transcripts that ultimately affect the outcome of DHAV infection. Transcripts with altered m6A modification during DHAV infection encode proteins may influence the outcome of infection with either proviral or antiviral effects, as previously reported (Gokhale et al., 2016; Lichinchi G, 2016; 11). Our comparison of the changes in the m6A methylation signals on cellular mRNAs in duckling liver upon infection by virulent and attenuated DHAV demonstrated that many virulence-regulated m6A genes are involved in virus-induced pathways, including the viral defence response and immune response. These key biological processes are reconstituted by the vaccine strain and may support host immune defence given the importance of translation in antiviral gene expression (41–44).

In summary, we identified the presence of m6A modification motifs in the duckling liver along with unique motifs. Importantly, global m6A changes in transcripts are associated with DHAV virulence. The virulence-regulated global m6A changes in transcripts are associated with viral response and coupled with changes in mRNA levels. It could be hypothesized that the widespread distribution of m6A throughout the duck transcriptome would afford m6A roles in various aspects of RNA biology, including mRNA translation (45). Collectively, our work indicates that posttranscriptional regulation of specific transcripts by m6A is associated with the virulence of a virus and induce different viral responses by contributing to immune-regulated gene expression during viral infection.

## Materials and Methods

### Viruses and Animals

The virulent DHAV-1 CH strain and the attenuated DHAV-1 CH60 strain were provided by the Institute of Preventive Veterinary Medicine, Sichuan Agricultural University. Ducklings were infected with the CH strain at a concentration of 10^7.88^ copies/ml and the CH60 strain at a concentration of 10^8.07^ copies/ml as determined by qRT–PCR. One-day-old Cherry Valley ducks were purchased from the poultry farm of Sichuan Agricultural University and were raised in isolators. The ducks were confirmed to be free of DHAV-1.

### Viral Infection and Tissue Collection

After one week, the ducks were randomly divided into three groups with 15 ducks in each group and raised in separate isolators. The ducks in the first group received 0.40 ml of the DHAV-CH strain (10^7.88^ copies/ml) *via* intramuscular injection, the ducks in the second group received 0.25 ml of the DHAV-CH60 strain (10^8.07^ copies/ml) *via* intramuscular injection, and ducks in the last group were injected with 0.25 ml of 0.75% physiological normal saline (NS) as a negative control. Three ducklings from each group were sacrificed at 12, 24, 36, 48, 60, and 72 hpi, and their livers and blood were collected. Fifty-milligram liver specimens were weighed and immediately placed in a solution to protect the RNA and DNA in the samples (code. no 9750, TaKaRa, Japan) until RNA isolation was performed.

To identify the mortality rates of CH- and CH60-inoculated ducklings, 30 one-week-old ducks were randomly divided into three groups (n = 10) and raised in separate isolators. The ducks in the first group received 0.40 ml of the DHAV-CH strain (10^7.88^ copies/ml) *via* intramuscular injection, the ducks in the second group received 0.25 ml of the DHAV-CH60 strain (10^8.07^ copies/ml) *via* intramuscular injection, and the ducks in the last group were injected with 0.25 ml of 0.75% physiological normal saline (NS) as a negative control. Signs of disease and death were observed within one week.

### m6A Dot Blot Assay

Total RNA was extracted using TRIzol Reagent (Ambion) following the manufacturer’s instructions, and mRNA was subsequently purified using a GenElute™ mRNA Miniprep Kit (Sigma, MRN10). The m6A-dot blot was performed on the Bio-Dot^®^ Microfiltration Apparatus (170-6545, GE Healthcare) using Amersham Hybond-N+membrane (GE Amersham, RPN303B) in twofold dilutions. After UV cross-linking, the blotted membrane was washed with 1× PBST buffer, blocked with 5% nonfat milk, and incubated with primary rabbit anti-m6A antibody (Synaptic Systems, cat. 202003) overnight at 4°C. After incubation with horseradish peroxidase (HRP)-conjugated anti-rabbit IgG (DakoCytomation, p0448) secondary antibody, the membrane was visualized using a SuperSignal West Pico ECL substrate box (Thermo Pierce, 34087).

### Quantitative Analysis of m6A Levels Using LC–MS/MS

Quantification of m6A and m6Am in polyadenylated RNA was performed using LC–MS/MS as described previously (46). In brief, polyadenylated RNA was purified from total RNA using the GenElute™ mRNA Miniprep Kit (Sigma, MRN10) according to the manufacturer’s instructions. Then, 2 U nuclease P1 (Sigma–Aldrich, USA) was used to digest the polyadenylated RNA (approximately 200 ng) at 37°C for 2 h in 25 µl buffer (25 mM NaCl and 2.5 mM ZnCl_2_). After adding NH_4_HCO_3_ (1 M, 3 µl) and alkaline phosphatase (0.5 U), the reaction was maintained at 37°C for 2 h. Deionized water was added to dilute the digestion mixture to 1 ml. The diluted digestion mixture was centrifuged at 10,000 rpm for 5 min and filtered with a 0.22-µM Millipore membrane to remove any solid material. The solution was used for the LC–MS/MS analysis. The quantification was carried out using a standard curve generated from A, m6A and m6Am standards (0.1-10 nM for m6A and m6Am, 50-2000 nM for A) run during the same batch of samples. m6A and m6Am levels were calculated as the ratio of m6A and m6Am, respectively, to A.

### m6A Immunoprecipitation and m6A-Seq

The polyadenylated mRNAs isolated from total RNA using the GenElute™ mRNA Miniprep Kit (Sigma, MRN10) were fragmented into 100-nt lengths using RNA fragmentation buffer (0.1 M Tris-HCL, pH 7.0 and 0.1M ZnCl_2_). Then, 50 and 100 ng fragmented mRNAs were incubated for 2 hours at 4°C with 12.5 μg of anti-m6A antibody (Synaptic Systems, 202003) in IP buffer (0.05 M Tris-HCl pH 7.4, 0.375 M NaCl, 0.5% Igepal CA-630). The mixture was then subjected to immunoprecipitation by incubation with Pierce™ ChIP-grade Protein A/G Magnetic Beads (Thermo, 26162) at 4°C for 2 hours. After sufficient washing, m6A antibody-bound RNA was eluted from the beads with elution buffer (1× IP buffer, 7 mM m6A, RNase inhibitor) and then ethanol-precipitated. The eluted RNA was resuspended in H_2_O and used to generate the cDNA library according to the RNA-Seq Library Preparation Kit for Transcriptome Discovery-Illumina Compatible. Then, the library was sequenced using the HiSeq 2000 system (Illumina) according to the manufacturer’s instructions.

### Analysis of m6A-Seq Data

For m6A-Seq data, adaptors and low-quality bases were trimmed from raw sequencing reads using CutAdapt, and reads less than 16 nt were discarded. After quality control and data filtering, reads were aligned to the reference genome. To assess the m6A level of each gene, only reads unambiguously aligned were preserved to calculate read number and RPKM value (RPKM represents reads per kilobase and per million). To identify the m6A regions (statistically significant m6A peaks), we called m6A peaks from m6A-seq data by running the ABLIRC pipeline.

### Differentially Demethylated Genes (DMG)

Differentially demethylated genes between the paired groups were analyzed using edgeR in R packages for each gene, a P value of significance was obtained based on the model of the negative binomial distribution. Fold changes in gene expression were also estimated within the edgeR statistical package. The criterion for DMG was set as a fold change >2 or <0.5 and P < 0.01.

### Statistical Analysis

Experiments were performed at least thrice and representative results are shown. Differences between individual groups were analyzed using Student’s *t* test (two-tailed and unpaired) with triplicate or quadruplicate sets. A value of *P* < 0.05 was considered statistically significant.

## Data Availability Statement

The datasets presented in this study can be found in online repositories. The names of the repository/repositories and accession number(s) can be found below: https://www.ncbi.nlm.nih.gov/geo/query/acc.cgi?acc=GSE163905.

## Ethics Statement

The animal study was reviewed and approved by the Committee of Experiment Operational Guidelines and Animal Welfare of Sichuan Agricultural University.

## Author Contributions

LW, WQ, YZ, SM, MW, XO, QY, and AC designed the experiments. AC and MW contributed materials and experimental platforms. SM, XO, and DS acquired data. SM, XO, DS, ML, QY, YW, YXW, RJ, SC, DZ, ML, XZ, SZ, JH, QG, BT, and AC analyzed and interpreted data. SM and XO wrote the manuscript. SM, XO, DZ, SC, RJ, XZ, and ML proofread the draft. All authors approved the final version of the manuscript.

## Funding

This study was supported by grants from National Natural Science Foundation of China (32102706), Supported by China Agriculture Research System of MOF and MARA, and the Sichuan Veterinary Medicine and Drug Innovation Group of China Agricultural Research System (SCCXTD-2020-18).

## Conflict of Interest

WQ, YZ, and YXW were employed by ABLife Inc.

The remaining authors declare that the research was conducted in the absence of any commercial or financial relationships that could be construed as a potential conflict of interest.

## Publisher’s Note

All claims expressed in this article are solely those of the authors and do not necessarily represent those of their affiliated organizations, or those of the publisher, the editors and the reviewers. Any product that may be evaluated in this article, or claim that may be made by its manufacturer, is not guaranteed or endorsed by the publisher.
