# Supplementary material for: Attenuated Duck Hepatitis A Virus Infection Is Associated With High mRNA Maintenance in Duckling Liver via m6A Modification
Source: Front Immunol. 2022 Jun 9;13:839677. doi: 10.3389/fimmu.2022.839677 (PMC9218207; doi:10.3389/fimmu.2022.839677)
Supplement: Supplementary file 1 [file Presentation_1.pdf]

Supplementary Material

Attenuated duck hepatitis A virus infection is associated with high mRNA maintenance in duckling liver via m6A modification

Liping Wu, Weili Quan, Yi Zhang, Mingshu Wang, Xumin Ou, Sai Mao, Di Sun, Qiao Yang, Ying Wu, Yaxun Wei, Renyong Jia1, Shun Chen, Dekang Zhu, Mafeng Liu, Xinxin Zhao, Shaqiu Zhang, Juan Huang, Qun Gao, Bin Tian and Anchun Cheng

Correspondence： Anchun Cheng (chenganchun@vip.163.com)

This file includes：

Supplementary Figure 1-7

Supplementary Table 1-2

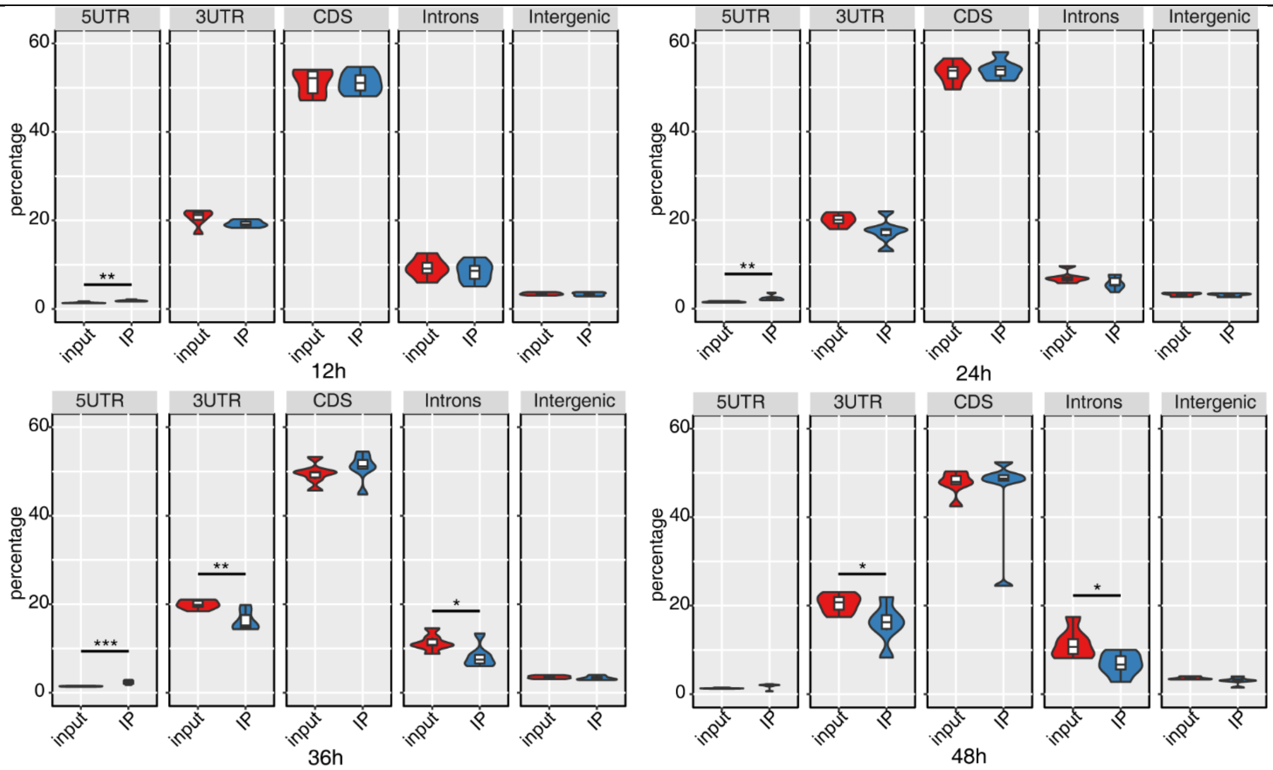

Supplementary Figure 1. Common features of m6A peak distributions among different gene regions. Data from RNA-seq (input) and m6A-seq (IP) of both DHAV CH60 strain or CH strain infected duckling livers were used. A similar analysis from different infection time points was presented.

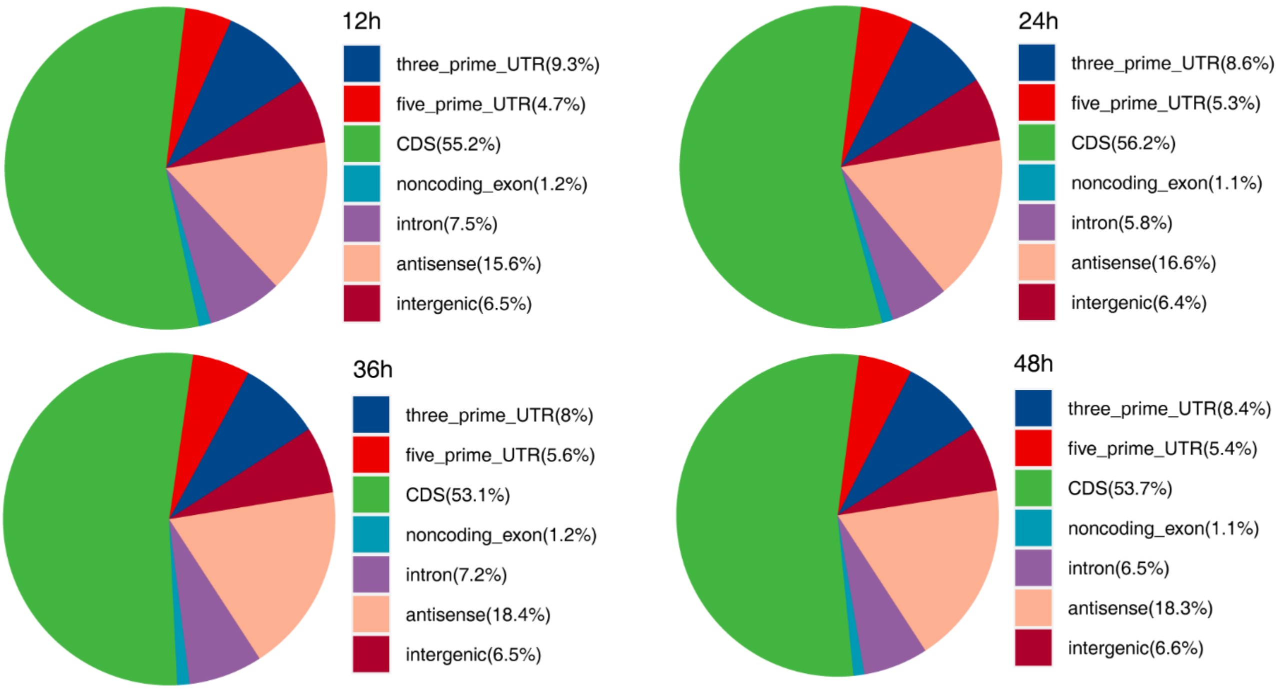

Supplementary Figure 2. m6A modified RNA mapping to different genomic regions. A similar analysis from different infection time points was presented. Besides the CDS region, m6A modification in the antisense region represent a large portion, a percentage from 15.6% to 18.4%. Data from RNA-seq (input) and m6A-seq (IP) of both DHAV CH60 strain or CH strain infected duckling livers were used.

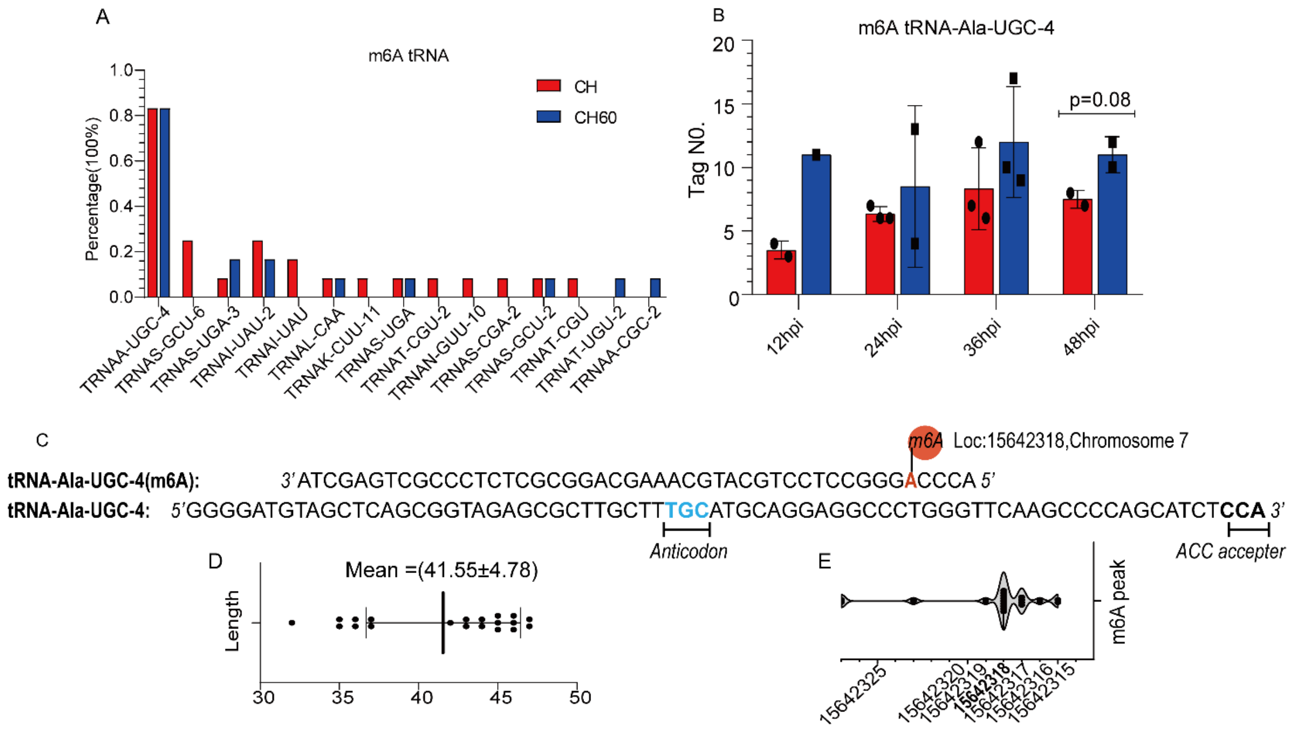

Supplementary Figure 3. M6A modification on tRNA-derived fragments. (**A**) tRNA-Ala-UGC-4 antisense strand-derived fragments were highly enriched during the infection. (**B**) The attenuated CH60 strain-induced higher expression of tRNA-Ala-UGC-4 antisense fragments than that of the virulent CH strain during infection. (**C**) binding of tRNA-Ala-UGC-4 antisense fragments to the tRNA-Ala-UGC-4. The m6A modification site and locations were indicated. (**D**) statistic length of tRNA-Ala-UGC-4 antisense fragments (41.55±4.78). (**E**) statistic m6A modification site at tRNA-Ala-UGC-4 antisense fragments (loc: 15642318, chromosome 7).

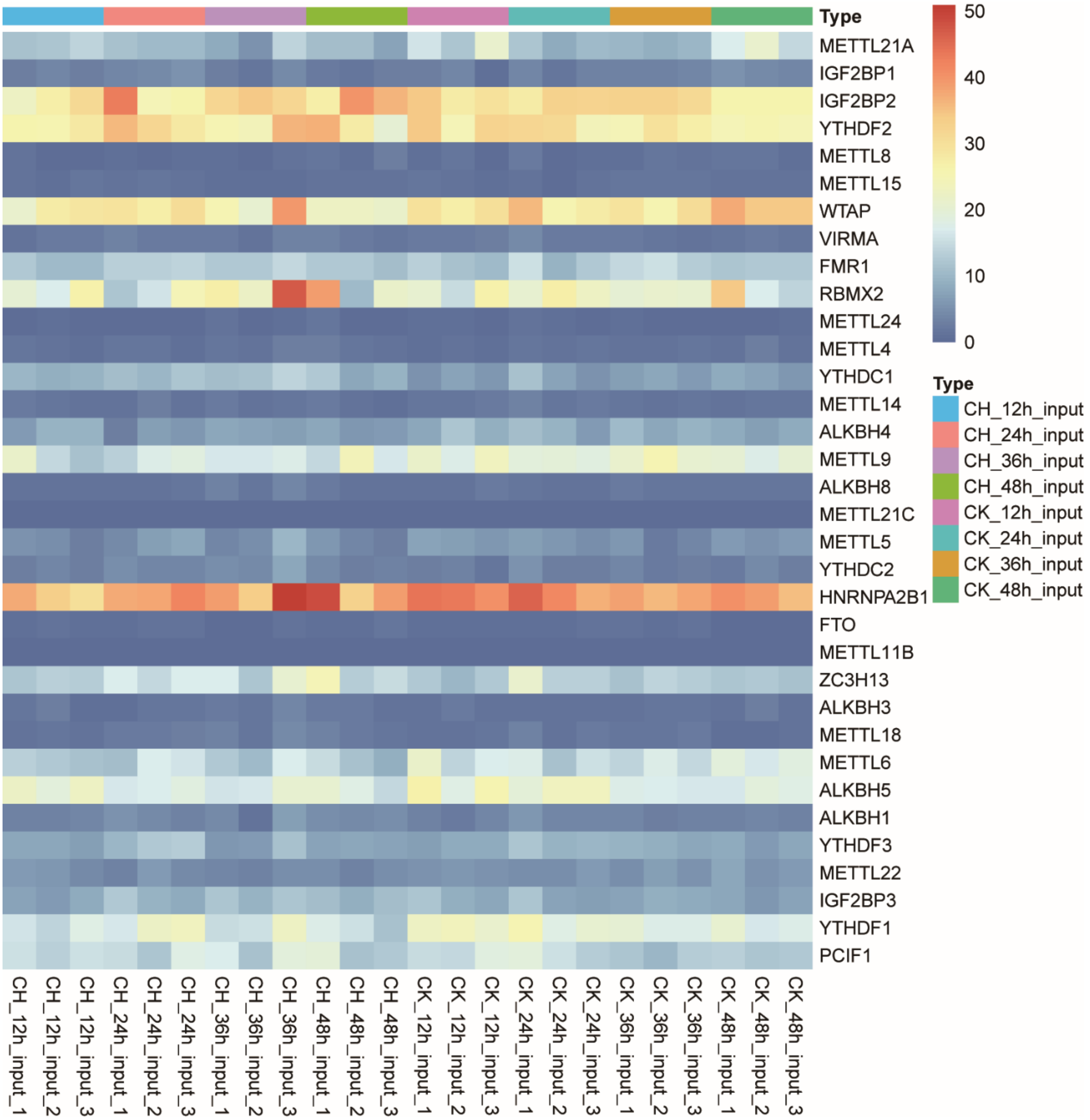

Supplementary Figure 4. The expression of potential m6A writers, erasers and readers in m6A-seq input samples, which were polyadenylated RNAs. Normalized FPKM values of each gene were presented.

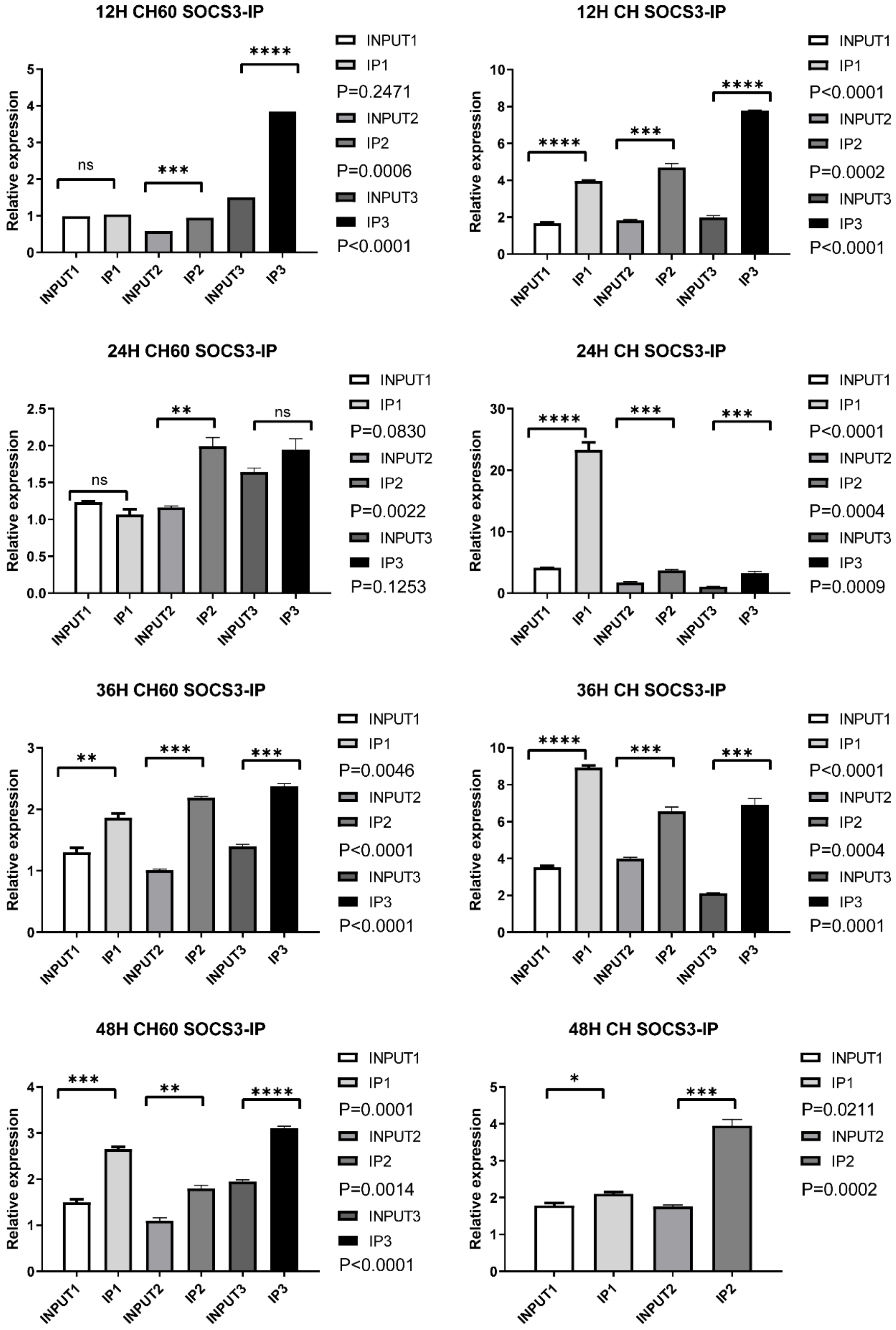

Supplementary Figure 5. Validation of m6A modified SOCS3 transcript by qRT-PCR method. Total input mRNA and m6A antibody immunoprecipitated mRNA were quantified and normalized by a

reference gene. SOCS3, suppressor of cytokine signaling 3.

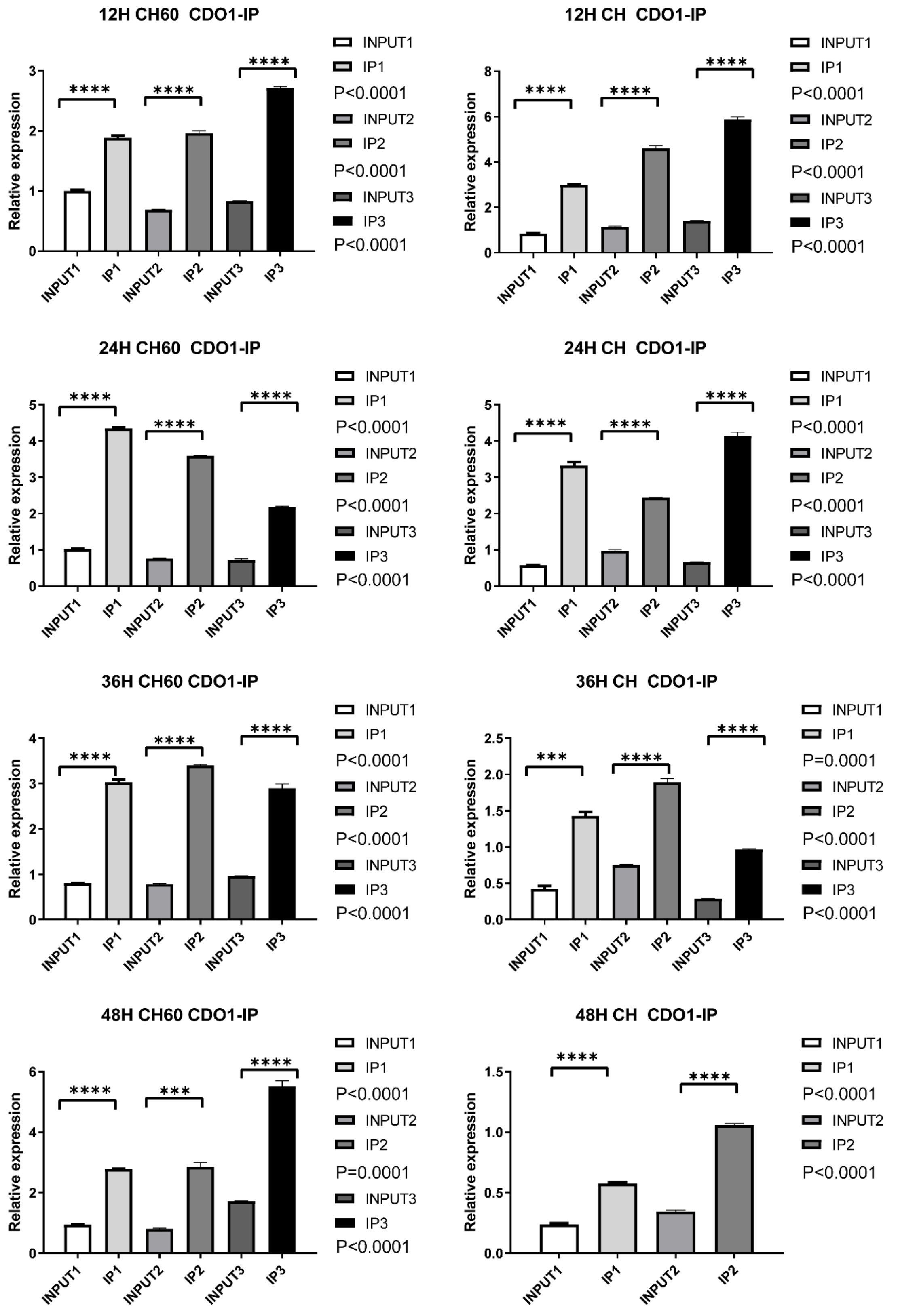

Supplementary Figure 6. Validation of m6A modified CDO1 transcript by qRT-PCR method. Total input mRNA and m6A antibody immunoprecipitated mRNA were quantified and normalized by a reference gene. CDO1, cysteine Dioxygenase Type 1.

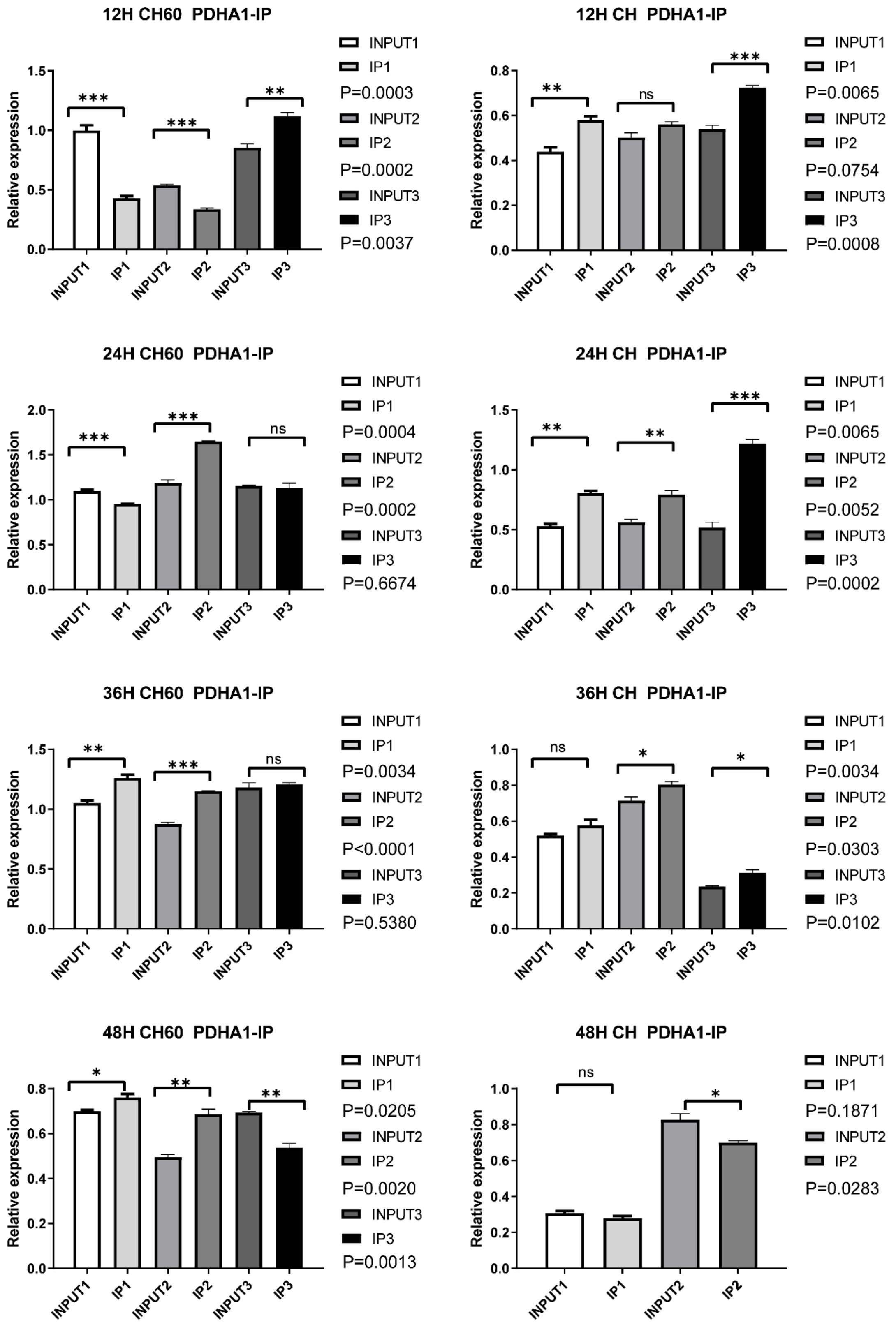

Supplementary Figure 7. Validation of m6A modified PDHA1 transcript by qRT-PCR method. Total input mRNA and m6A antibody immunoprecipitated mRNA were quantified and normalized by a reference gene. PDHA1, Pyruvate Dehydrogenase E1 Subunit Alpha 1.

Supplementary Table 1. MeRIP-seq generated in this study

SampleID	raw_total	clean_total	raw_base	clean_base	Total mapped	Total Uniquely mapped
CH_12h_input_1	42692564	41118569	6.404G	5.187G	37052951(90.11%)	32667121(88.16%)
CH_12h_input_2	42069670	40545969	6.310G	5.084G	36545046(90.13%)	32162745(88.01%)
CH_12h_input_3	48319958	46591549	7.248G	5.802G	42022305(90.19%)	37224440(88.58%)
CH_12h_IP_1	41874488	40164264	6.281G	4.756G	36096344(89.87%)	31396870(86.98%)
CH_12h_IP_2	41378606	39331703	6.207G	4.679G	35328291(89.82%)	30729223(86.98%)
CH_12h_IP_3	45315548	43150417	6.797G	5.117G	38916668(90.19%)	34133851(87.71%)
CH_24h_input_1	47480258	46035282	7.122G	5.804G	41548736(90.25%)	36900382(88.81%)
CH_24h_input_2	46858488	44814323	7.029G	5.669G	40243966(89.8%)	35640639(88.56%)
CH_24h_input_3	47211060	45218568	7.082G	5.578G	40625667(89.84%)	35956910(88.51%)
CH_24h_IP_1	39229248	37778036	5.884G	4.203G	33826264(89.54%)	29184722(86.28%)
CH_24h_IP_2	42811614	37925232	6.422G	4.491G	34218973(90.23%)	29791585(87.06%)
CH_24h_IP_3	46863354	44565651	7.030G	5.225G	39945284(89.63%)	34769994(87.04%)
CH_36h_input_1	43029222	41346125	6.454G	5.022G	37421408(90.51%)	32656608(87.27%)
CH_36h_input_2	44022958	42624397	6.603G	5.419G	38149185(89.5%)	33564616(87.98%)
CH_36h_input_3	56409346	54507214	8.461G	6.631G	49256943(90.37%)	43180175(87.66%)
CH_36h_IP_1	38163028	36394418	5.724G	4.039G	32709030(89.87%)	28147083(86.05%)
CH_36h_IP_2	41399530	36501866	6.210G	4.107G	33126239(90.75%)	28078027(84.76%)
CH_36h_IP_3	49107818	47187858	7.366G	5.232G	42013803(89.04%)	35920567(85.5%)
CH_48h_input_1	46389464	44894420	6.958G	5.604G	40561016(90.35%)	35481159(87.48%)
CH_48h_input_2	47391274	45855701	7.109G	5.760G	42161835(91.94%)	36648783(86.92%)
CH_48h_input_3	53266168	51575822	7.990G	6.398G	47437961(91.98%)	41173414(86.79%)
CH_48h_IP_1	50723960	48851020	7.609G	5.365G	44152651(90.38%)	37582149(85.12%)
CH_48h_IP_2	41296318	38655560	6.194G	4.310G	35438547(91.68%)	29781726(84.04%)
CH_48h_IP_3	29659000	811199	4.449G	0.085G	113868(14.04%)	98415(86.43%)
CK_12h_input_1	53470988	51603766	8.021G	6.343G	47280740(91.62%)	41523009(87.82%)
CK_12h_input_2	47605116	45893590	7.141G	5.631G	41984421(91.48%)	36999967(88.13%)
CK_12h_input_3	47574940	45963167	7.136G	5.661G	41447333(90.18%)	36557097(88.2%)
CK_12h_IP_1	38894260	35960528	5.834G	4.245G	32436543(90.2%)	28238548(87.06%)
CK_12h_IP_2	38980936	36877408	5.847G	4.281G	33201772(90.03%)	28966925(87.25%)
CK_12h_IP_3	48893670	46665619	7.334G	5.367G	42060422(90.13%)	36393733(86.53%)
CK_24h_input_1	44740396	43131140	6.711G	5.432G	38828971(90.03%)	34799337(89.62%)
CK_24h_input_2	43498874	41952147	6.525G	5.176G	37918913(90.39%)	33586406(88.57%)
CK_24h_input_3	32622258	31612985	4.893G	3.950G	28602338(90.48%)	25446667(88.97%)
CK_24h_IP_1	42034550	39402469	6.305G	4.688G	35231081(89.41%)	31313002(88.88%)
CK_24h_IP_2	39932650	36833385	5.990G	4.312G	32829736(89.13%)	28596332(87.1%)
CK_24h_IP_3	42499602	40823906	6.375G	4.374G	37037695(90.73%)	31713887(85.63%)
CK_36h_input_1	49424832	47330121	7.414G	5.930G	42635260(90.08%)	37473241(87.89%)
CK_36h_input_2	49734466	47626475	7.460G	5.894G	43065107(90.42%)	37936551(88.09%)
CK_36h_input_3	41202626	39532459	6.180G	4.885G	36006716(91.08%)	31480279(87.43%)
CK_36h_IP_1	47746594	45041555	7.162G	5.112G	40444422(89.79%)	34270543(84.73%)
CK_36h_IP_2	44097722	41612002	6.615G	4.768G	37264465(89.55%)	31909002(85.63%)
CK_36h_IP_3	40656834	37590538	6.099G	4.280G	33579098(89.33%)	28408603(84.6%)
CK_48h_input_1	49401410	47851464	7.410G	6.034G	43540362(90.99%)	38335103(88.04%)
CK_48h_input_2	44818846	43419446	6.723G	5.488G	39417871(90.78%)	34698249(88.03%)
CK_48h_input_3	46503788	45007565	6.976G	5.697G	40953035(90.99%)	36206729(88.41%)
CK_48h_IP_1	38120642	36321281	5.718G	4.234G	33083598(91.09%)	28265253(85.44%)

CK_48h_IP_2	46005930	43483885	6.901G	4.786G	39676373(91.24%)	33401719(84.19%)
CK_48h_IP_3	43624058	41915479	6.544G	4.740G	37695105(89.93%)	32451788(86.09%)

Supplementary Table 2. Primers used in the validation of m6A targeting genes.

Gene name	Primer (5'-3')
SOCS3-F	CCCAACACGACGGGCACGAG
SOCS3-R	AGCACAAGAGCAGGGGACAGG
PDHA1-F	GGCTTGAAGGAGACTCGAGGACGG
PDHA1-R	CCAGCAGCATCTTGCGCATGG
CDO1-F	CAGACGGAGATGTGGAAG
CDO1-R	GTTGCTCTCGTAGGACTC
Reference-F	AATTAACCCTCACTAAAGGG
Reference-R	GTCCTTATCATCGTCGTCTT
